# From Global Evidence to Saudi Practice: A Structured Evidence Map of the Diagnosis, Molecular Targets, and Therapeutic Strategies of Multiple Sclerosis in the Kingdom of Saudi Arabia

**DOI:** 10.3390/diseases14100382

**Published:** 2026-10-18

**Authors:** Rashid Mir, Naseh A. Algehainy, Mohammed M. Jalal, Malik A. Altayar, Reema M. Almotairi, Faris J. Tayeb, Ruqaiah I. Bedaiwi, Faisal H. Altemani, Kholoud S. Almasoudi, Ibrahim Altedlawi Albalawi, Eram Husain

**Affiliations:** 1 Prince Fahad Bin Sultan Chair for Biomedical Research, University of Tabuk, Tabuk 71491, Saudi Arabia; 2 Department of Medical Laboratory Technology, Faculty of Applied Medical Sciences, University of Tabuk, Tabuk 71491, Saudi Arabia; 3 Department of Surgical Oncology, Faculty of Medicine, University of Tabuk, Tabuk 47512, Saudi Arabia

**Keywords:** multiple sclerosis, Saudi Arabia, disease-modifying therapy, treatment access, health-system readiness, neuromyelitis optica spectrum disorder, vitamin D, research priorities

## Abstract

Multiple sclerosis (MS) has shifted in a generation from an uncommon diagnosis in Saudi Arabia to a major cause of neurological disability in young adults. Available Saudi data describe clinical characteristics broadly similar to those reported elsewhere in the Middle East and do not show that Saudi patients differ from trial populations, although this does not establish equivalent efficacy or safety. How far global advances are usable in the Kingdom has not, however, been synthesized. This narrative review maps diagnostic and therapeutic strategies across five separately reported domains (clinical evidence, 2022 national consensus listing, affordability, monitoring requirement, regional data), with categories assigned by a pre-specified rule, not a composite grade. Regional evidence on established therapies is entirely observational: cohorts report favourable outcomes with fingolimod, natalizumab, and off-label rituximab but cannot establish comparative effectiveness, and physicians report cost, availability, and insurance formularies as constraints whose extent has not been quantified. Diagnosis, now based on the 2024 McDonald revision, is transferable in knowledge but depends on AQP4-IgG testing, MRI, and biomarker capacity whose national distribution is undocumented, with neuromyelitis optica spectrum disorder an important differential. Tolebrutinib, recently approved in Europe for non-relapsing secondary progressive MS, and experimental modalities remain regulatory or research questions without regional data. Saudi studies document substantial payer costs of disease-modifying therapy, but none have measured how often cost or infrastructure alters treatment; we propose a prioritized research agenda.

## 1. Introduction

The evidence that underpins current multiple sclerosis care has been generated predominantly in European and North American populations, and Saudi neurologists, like their counterparts elsewhere, must judge how that evidence translates into local practice. For decades MS was considered uncommon in the Kingdom; an early community survey, the Thugbah study, recorded low counts that may have reflected limited case ascertainment as much as true rarity [1]. That picture has changed. Hospital-based series now describe a disease that is more common, and more often diagnosed, than earlier figures implied [2], mirroring a worldwide rise in recorded prevalence [3].

Clinical data from the Kingdom nonetheless remain limited. They come mainly from hospital-based series, whose authors describe characteristics similar to those reported elsewhere in the Middle East [2], and they do not show that Saudi patients differ from trial populations in ways that would alter treatment efficacy or safety. Section 3 examines these data, together with three issues often raised in the Saudi context: the distinction between MS and neuromyelitis optica spectrum disorder (NMOSD), consanguinity, and vitamin D status.

The global therapeutic menu, meanwhile, has expanded far beyond the injectable immunomodulators of twenty years ago. Oral sphingosine-1-phosphate modulators, high-efficacy anti-CD20 monoclonals, Bruton tyrosine kinase inhibitors, and a research pipeline reaching into natural killer (NK) cell modulation, CRISPR-based editing of autoreactive circuits, dopaminergic immunomodulation, plant-derived neuroprotectants, and exosome-based delivery now populate the literature. The problem for a Saudi clinician or investigator is not access to this information—it is knowing which of these advances is deployable in Riyadh or Dammam today, which is constrained by cost or infrastructure, and which remains a laboratory prospect with no regional footing at all.

Existing regional literature addresses this question only in fragments. Saudi and pan-regional expert panels have produced focused consensus documents—on management during the COVID-19 pandemic [4], on pregnancy and family planning [5], on pediatric and adolescent disease [6], and, at MENA scale, on NMOSD diagnosis and treatment [7]. A 2026 scoping review has mapped the genetic and molecular architecture of MS across Gulf Cooperation Council populations and argued, persuasively, that Western HLA-DRB1*15:01-centered susceptibility models may not transfer intact to Arabian Gulf ancestry [8]. What no synthesis has yet done is take the full therapeutic spectrum—from first-line disease-modifying therapy to the most speculative experimental modality—and ask, systematically and section by section, how each stands in relation to the Saudi context.

This review is built around that question. Rather than surveying global advances and appending occasional Saudi references, we map each diagnostic and therapeutic strategy across separately reported domains: clinical evidence and the locally reported clinical context; regulatory availability; affordability; monitoring requirement; and regional evidence. Where robust Saudi or Gulf evidence exists—as it does for disease-modifying therapy cost-effectiveness [9], prescribing behavior [10], and real-world outcomes with fingolimod, rituximab, and natalizumab [11,12,13]—we report it alongside the international evidence. Where such evidence is absent, as it is for most emerging modalities, we say so plainly and convert that absence into a defined regional research priority rather than treating it as evidence of reduced efficacy or suitability.

To the best of our knowledge, this is the first review to translate the full spectrum of established and emerging MS therapeutic strategies into a structured evidence map for the Saudi context, reporting clinical evidence, availability, affordability, monitoring requirements, and regional evidence separately, together with and a prioritized regional research agenda.

## 2. Search Strategy and Conceptual Framework

This is a narrative review. To make the selection of literature transparent, it was informed by a structured search with pre-specified databases, date limits, and search strings, but it did not follow a systematic-review protocol: no protocol was registered, and the review did not include duplicate independent screening, formal risk-of-bias assessment, or quantitative synthesis. This design was chosen because the evidence relevant to an applied regional question is heterogeneous, spanning randomized trials, observational cohorts, consensus statements, and mechanistic studies, and is not amenable to pooled analysis. Its principal limitation is that study selection, and the weight given to individual studies, involved author judgement, so the coverage may not be exhaustive. We searched PubMed, Scopus, and Web of Science for English-language publications from January 2000 to February 2026, with a verification pass immediately before submission and an update in October 2026 during revision to capture recent additions, including the 2024 revision of the McDonald criteria, Saudi cost studies, and product-label monitoring requirements. Search strings combined disease terms (“multiple sclerosis”, “demyelinating disease”) with, in one axis, geographic terms (“Saudi Arabia”, “Gulf”, “GCC”, “Arab”, “Middle East”), and in a second axis, the specific diagnostic and therapeutic topics examined here (for example “disease-modifying therapy”, “ocrelizumab”, “rituximab”, “fingolimod”, “natalizumab”, “NMOSD”, “vitamin D”, “CRISPR”, “exosome”). Reference lists of retrieved articles and the published Saudi Ministry of Health and MENA consensus statements were hand-searched for sources not indexed under these terms.

Priority was given to primary studies—original clinical cohorts, real-world registries, and mechanistic work—with review articles used mainly to frame background. Regional (Saudi and Gulf) sources were retained whenever they reported primary data relevant to any of the evidence-map domains defined below; global sources were retained when they established a mechanism, a pivotal trial result, or a landmark epidemiological finding that a regional judgment had to be measured against. Where an initial citation match was ambiguous, article metadata were checked against the source record before inclusion.

The organizing device of this review is a structured evidence map. For each strategy we report five domains separately and do not combine them into a single grade, because availability, affordability, and clinical suitability are distinct questions: a treatment may be available yet unaffordable for some patients, or may lack regional studies yet remain clinically appropriate. (i) Clinical suitability: the pivotal evidence and approved indication, together with what Saudi data report about the clinical context in which the strategy would be used (for example, the disease course at diagnosis or the need to exclude NMOSD). Trial-derived estimates of efficacy and safety are used as the best available estimates for Saudi patients. This is a working assumption, not a demonstration of equivalence: the absence of evidence of a difference does not establish that efficacy and safety are the same, and we identified no Saudi-specific subgroup analyses of the pivotal trials. This follows the ICH E5(R1) principle that clinical data generated in one region can be extrapolated to another when a medicine is not sensitive to ethnic factors and extrinsic factors such as medical practice are similar [14]. The same default underlies Saudi drug regulation: the SFDA verification and abridged pathways register medicines on the basis of FDA and EMA assessments, provided that the product “does not need a more stringent assessment as a result of different local disease patterns and/or medical practices” [15]. Where regional data exist, the assumption is tested rather than merely asserted: relapse outcomes in regional observational cohorts of fingolimod and rituximab are consistent with pivotal-trial results (Section 6 and Section 8) [11,16]. Where Saudi data document a population factor that could modify benefit–risk, it is reported against the relevant drug, for example the CYP2C9 genotype that determines siponimod eligibility and dose [17,18] and the anti-JC virus seroprevalence relevant to natalizumab [19] (Table 1). Confirming transferability is listed as a research priority. (ii) National consensus listing: whether the strategy is listed as a treatment option in the 2022 Saudi consensus recommendations on disease-modifying therapy [20], is used off-label, or awaits a Saudi regulatory decision. This domain records national clinical endorsement at a fixed date and is not a measure of current availability: inclusion in the consensus does not establish that a drug is currently registered, supplied, included in formularies, or reimbursed, and agents approved internationally after 2022 may since have become available. We used the consensus because it is the only peer-reviewed, nationally authored source that lists disease-modifying options for the Kingdom; the SFDA public drug list reports trade names, strengths, dosage forms, and prices but not formulary or reimbursement status [21], and Saudi neurologists report that drug availability differs between hospitals and is restricted by institutional formularies [10]. Current national availability is therefore treated as unknown, and its measurement is listed as a research priority. (iii) Affordability: whether Saudi cost, cost-effectiveness, or reimbursement data exist, and what they show. (iv) Monitoring requirement: the safety-monitoring and delivery services required by the product label, graded by the rule given below. (v) Regional evidence: whether Saudi or Gulf data exist. This last domain is descriptive only; the absence of regional studies is reported as a research gap and is not used to infer lower efficacy, safety, or suitability for Saudi patients.

**Figure 1:**
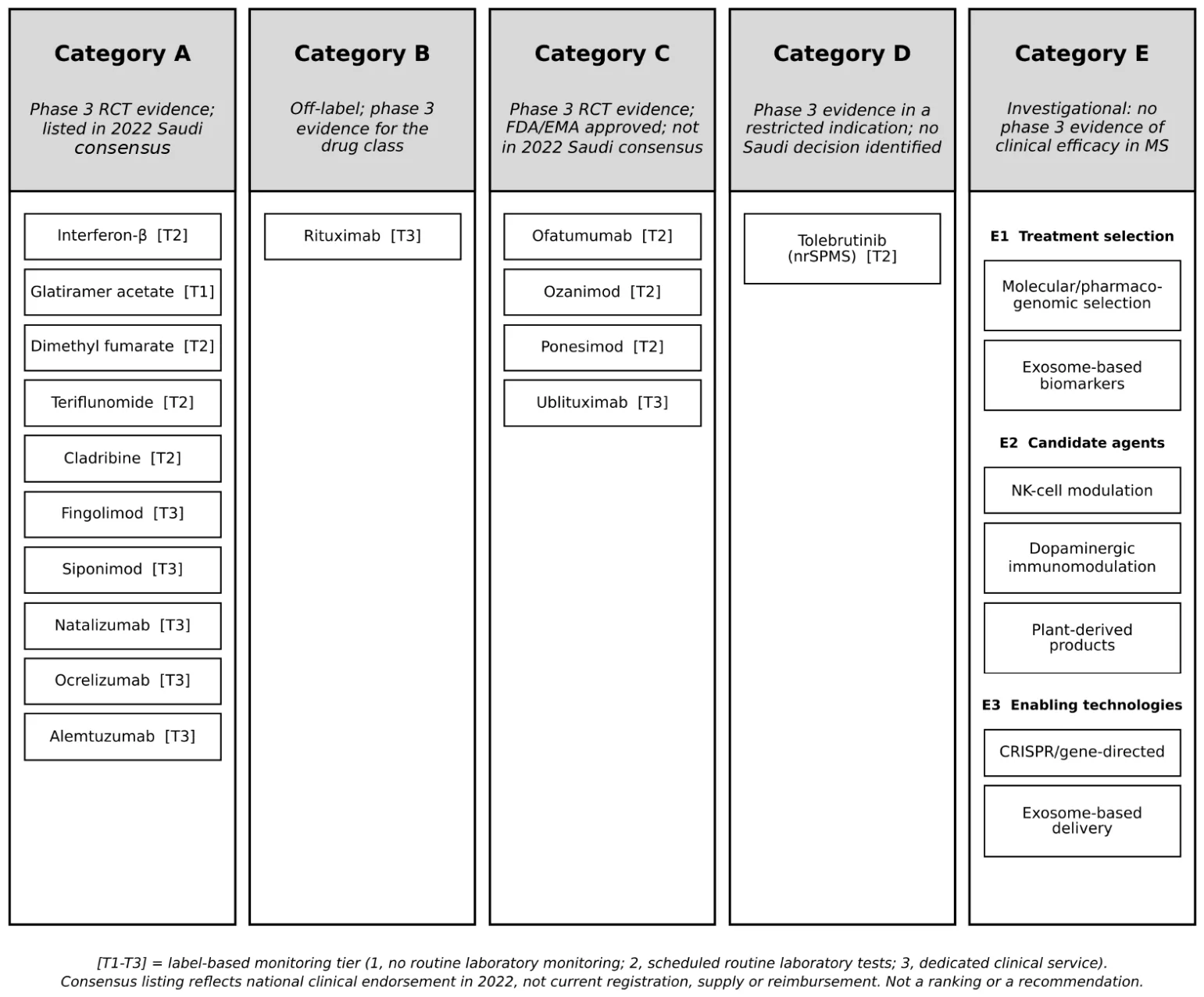
Evidence-status categories for therapeutic strategies in the Saudi MS setting, assigned by the pre-specified rule in Section 2. Categories use only clinical evidence and Saudi availability: (A) phase 3 randomized evidence and listed as a treatment option in the Saudi consensus recommendations [20]; (B) off-label use with phase 3 evidence for the drug class; (C) phase 3 randomized evidence and FDA or EMA approval but not listed in the 2022 Saudi consensus (such agents may have been registered since); (D) phase 3 evidence in a restricted indication with no Saudi regulatory decision identified; (E) investigational, with no phase 3 evidence of clinical efficacy in MS, subdivided by purpose into treatment-selection approaches (E1), candidate therapeutic agents (E2), and enabling technologies (E3). Consensus listing reflects national clinical endorsement in 2022, not current registration, supply, formulary inclusion, or reimbursement. The label-based monitoring tier (T1, no routine laboratory monitoring; T2, scheduled routine laboratory tests; T3, dedicated clinical service) is shown beside each therapy for information and does not determine the category; affordability evidence and regional data are reported separately in Table 1. Categories are not a ranking of efficacy and are not treatment recommendations; diagnosis is not categorized (Section 4).

Each therapeutic strategy is assigned to one evidence-status category by a pre-specified rule that uses only the first two domains: (A) phase 3 randomized evidence for the relevant MS indication and listed as a treatment option in the Saudi consensus recommendations [20]; (B) off-label use, with phase 3 randomized evidence for the drug class but not for the agent in its licensed indication, as listed in the same consensus; (C) phase 3 randomized evidence and approval by the US Food and Drug Administration or the European Medicines Agency, but not listed among the options in the 2022 Saudi consensus [20] (such agents may have been registered in the Kingdom since 2022, which we did not assess); (D) phase 3 evidence confined to a restricted indication, with no Saudi regulatory decision identified; and (E) investigational, with no phase 3 evidence of clinical efficacy in MS. Because category E is defined by evidence status, it brings together approaches with different purposes, and it is therefore subdivided by purpose: (E1) treatment-selection approaches, which would guide the choice of existing therapies (molecular or pharmacogenomic selection, exosome-based biomarkers); (E2) candidate therapeutic agents or immunomodulatory strategies (NK-cell modulation, dopaminergic immunomodulation, plant-derived compounds); and (E3) enabling technologies, which are at present research tools or delivery platforms rather than treatments (CRISPR-based editing, exosome-based delivery). Affordability, monitoring requirement, and regional evidence do not move a strategy between categories; they are reported alongside it in Table 1. The monitoring requirement is graded from the product label: tier 1, no routine laboratory monitoring is mandated; tier 2, scheduled tests available in any hospital laboratory (for example, blood counts or liver tests), with or without pre-treatment screening; tier 3, a dedicated clinical service beyond routine laboratory testing is required, such as an infusion unit, observed first-dose cardiac monitoring, specialized genotyping or serological risk stratification, or structured long-term post-treatment surveillance. Diagnosis is mapped across the same domains but is not assigned a therapeutic category. Table 1 sets out the domains for each strategy and Figure 1 shows the resulting categories; neither implies a ranking of therapies.

Scope and intended use. The evidence map is not a clinical practice guideline and makes no treatment recommendations. Treatment selection for individual patients in the Kingdom is governed by Saudi consensus recommendations developed by Saudi MS neurologists [6,20,22] and by regional MENACTRIMS guidance [7,23], to which we defer on all clinical questions. The purpose of the map is narrower: to show, for each strategy, which kinds of evidence exist for the Saudi setting and which are missing, so that missing evidence can be converted into research priorities. The author team is drawn mainly from laboratory medicine and biomedical research; this expertise bears most directly on the diagnostic and monitoring domains (AQP4-IgG cell-based assays, JC virus serology, lymphocyte and liver monitoring, and biobanking), and the clinical domains are taken from published trials, product labels, and Saudi consensus documents rather than from author judgement.

**Table 1:** Evidence map of diagnostic and therapeutic strategies for the Saudi MS setting: each domain is reported separately and no composite grade is assigned.

Strategy [Category]	Clinical Suitability (Pivotal Evidence; Indication)	Listing in 2022 Saudi Consensus (Not Current Availability)	Saudi Affordability Evidence	Monitoring Requirement (Label-Based Tier)	Regional (Saudi/Gulf) Data (Descriptive)
Diagnosis: 2024 McDonald criteria + AQP4-IgG cell-based assay [not categorized]	2024 revision is the current international standard [24]; NMOSD must be excluded before DMT [7,25]	Saudi MoH diagnostic consensus based on 2017 revision [22]; national distribution of AQP4-IgG assays, MRI and newer biomarkers not documented	No Saudi data	MRI and cell-based AQP4-IgG assay; 2024 adjuncts (central vein sign, paramagnetic rim lesions, CSF kappa free light chains, optic nerve assessment) need additional capacity	Saudi diagnostic consensus [22]; MENACTRIMS NMOSD guidance [7]
Interferon-β [A]	PRISMS: relapse reduction vs. placebo [26]; modest efficacy	Listed in Saudi consensus [20]	Modelled as not cost-effective at USD 100,000/QALY [27]; costlier than oral agents in one Riyadh centre [9]	Tier 2: liver tests and blood counts at 1, 3 and 6 months, then periodically; thyroid tests; depression surveillance [28]	Long regional use; dominated registry prescribing 2015–2018 [10]
Glatiramer acetate [A]	Copolymer-1 trial: fewer relapses vs. placebo [29]	Listed in Saudi consensus [20]	No agent-specific Saudi cost data	Tier 1: no routine laboratory monitoring; injection-site reactions, lipoatrophy; anaphylaxis boxed warning [30]	No agent-specific Saudi outcome data identified
Dimethyl fumarate [A]	DEFINE: relapse and MRI benefit [31]	Listed in Saudi consensus [20]	Modelled [27]; oral class less costly than interferon in one centre [9]	Tier 2: blood count with lymphocytes before treatment, at 6 months, then every 6–12 months; liver tests; PML risk with lymphopenia [32]	Included in pooled Saudi class analysis only [9]
Teriflunomide [A]	TEMSO: reduced relapse rate and disability [33]	Listed in Saudi consensus [20]	Modelled [27]; oral class less costly than interferon in one centre [9]	Tier 2: ALT at least monthly for 6 months; blood count; latent TB screening; blood pressure; pregnancy exclusion, contraception, accelerated elimination [34]	Included in pooled Saudi class analysis only [9]
Fingolimod [A]	FREEDOMS: ARR 0.18 vs. 0.40 [35]	Listed in Saudi consensus [20]	Modelled [27]; pooled oral class [9]	Tier 3: observed first-dose cardiac monitoring; macular assessment; VZV serology	FINOMENA, MENA incl. Saudi sites, *n* = 806 [11]
Siponimod [A]	EXPAND: reduced disability progression in SPMS [36]	Listed in Saudi consensus [20]	No Saudi cost data	Tier 3: CYP2C9 genotyping before treatment (*3/*3 contraindicated; reduced maintenance dose for *1/*3 and *2/*3) [17]; cardiac and macular assessment	No Saudi/Gulf outcome data; CYP2C9*3 allele frequency 5.3% in 11,889 Saudi individuals [18]
Ozanimod, ponesimod [C]	RADIANCE, SUNBEAM: lower ARR vs. interferon beta-1a [37,38]; OPTIMUM: lower ARR vs. teriflunomide [39]	Not listed in 2022 Saudi consensus [20]; post-2022 registration not assessed	No Saudi cost data	Tier 2: baseline ECG and dose titration (ponesimod titrated to mitigate first-dose cardiac effects [39]); blood counts, liver tests	No Saudi/Gulf data identified
Cladribine [A]	CLARITY: reduced relapse and MRI activity [40]	Listed in Saudi consensus [20]; SFDA registration 2021 [41]	Manufacturer-supported Saudi payer model [42]	Tier 2: lymphocyte counts around each treatment course; infection screening	No Saudi outcome data; lowest monitoring burden in Saudi Delphi [41]
Ocrelizumab [A]	OPERA I/II vs. interferon beta-1a [43]	Listed in Saudi consensus [20]	Two-centre Riyadh cost-consequence data [44]	Tier 3: infusion unit; immunoglobulins, infections	MENACTRIMS comparative registry, 509 on ocrelizumab [13]
Ofatumumab [C]	ASCLEPIOS I/II vs. teriflunomide [45]	Not listed in 2022 consensus; post-2022 registration not assessed; positioned for Saudi practice by an expert panel [46]	No Saudi cost data; comparative cost data called for [46]	Tier 2: self-injection; hepatitis B screening; immunoglobulins, infections	Expert-panel analysis only [46]
Ublituximab [C]	ULTIMATE I and II: lower ARR vs. teriflunomide [47]	Not listed in 2022 consensus (EU authorisation May 2023 [48]); post-2022 registration not assessed	No Saudi cost data	Tier 3: intravenous infusion; infusion-related reactions in 47.7% in ULTIMATE [47]	No Saudi/Gulf data identified
Rituximab [B]	Phase 3 evidence for the anti-CD20 class [43,45]; agent-specific evidence observational	Off-label option in Saudi consensus [20]; <1% of registry prescriptions 2015–2018 [10]	Less costly than natalizumab in two Riyadh centres [44]	Tier 3: infusion unit; immunoglobulins, infusion reactions, infections	Saudi *n* = 102 [12]; MENACTRIMS *n* = 774 [16] and comparative *n* = 1954 [13]
Natalizumab [A]	AFFIRM: high efficacy [49]	Listed in Saudi consensus [20]	Incremental cost about USD 35,383 vs. rituximab [44]	Tier 3: infusion unit; JC virus antibody-index risk stratification [50], switch advised at index > 0.9 [20]; PML vigilance [51]	MENACTRIMS comparative registry, 677 on natalizumab [13]; anti-JCV seropositivity 39.6% and pre-treatment testing in 4 of 83 treated patients at one Riyadh centre [19]
Alemtuzumab [A]	CARE-MS I and II [52,53]	Listed in Saudi consensus as escalation in aggressive MS [20]	No Saudi cost data	Tier 3: infusion; structured long-term autoimmunity surveillance	Highest monitoring burden in Saudi Delphi [41]
Tolebrutinib [D]	HERCULES: reduced disability progression in nrSPMS [54]; GEMINI: not superior to teriflunomide in relapsing MS [55]	EU- and UAE-approved for nrSPMS; no Saudi decision identified [56]	No Saudi cost data	Tier 2: intensive early liver-test schedule [57]	No Saudi/Gulf data
Molecular-target/pharmacogenomic selection [E1]	Not clinically validated; Western HLA model may not transfer to Gulf ancestry [8]	Not applicable	Not applicable	Would require high-resolution HLA/non-HLA genotyping	Fragmented GCC evidence [8]
NK-cell modulation [E2]	Investigational [58]	Not applicable	Not applicable	Would require immunophenotyping capacity	No regional data
CRISPR/gene-directed strategies [E3]	Laboratory tool only [59]	Not applicable	Not applicable	Would require validated regional genomics	No regional data
Dopaminergic immunomodulation [E2]	Preclinical [60]	Not applicable	Not applicable	Exploratory	No regional data
Plant-derived/natural products [E2]	Experimental models only [61]	Not applicable	Not applicable	Herb–drug interaction pharmacovigilance needed	No controlled Saudi trials; use alongside DMTs not quantified
Exosome-based biomarkers [E1]/delivery [E3]	Experimental [62]	Not applicable	Not applicable	Would require biobanking and standardized sample handling	No regional data

Category letters follow the pre-specified rule in Section 2: A, phase 3 randomized evidence and listed in Saudi consensus [20]; B, off-label with class evidence; C, phase 3 evidence and FDA/EMA approval but not in the 2022 Saudi consensus (may have been registered since); D, phase 3 evidence in a restricted indication, no Saudi decision identified; E, investigational (E1, treatment-selection approaches; E2, candidate therapeutic agents; E3, enabling technologies). The consensus-listing column records national clinical endorsement in 2022, not current registration, supply, formulary inclusion, or reimbursement. Categories do not reflect affordability, monitoring, or regional evidence and are not a ranking. Monitoring tiers: 1, no routine laboratory monitoring mandated; 2, scheduled routine laboratory tests; 3, dedicated clinical service required. Abbreviations: ALT, alanine aminotransferase; AQP4, aquaporin-4; ARR, annualized relapse rate; CSF, cerebrospinal fluid; DMT, disease-modifying therapy; EMA, European Medicines Agency; FDA, US Food and Drug Administration; GCC, Gulf Cooperation Council; JCV, JC virus; MoH, Ministry of Health; nrSPMS, non-relapsing secondary progressive MS; PML, progressive multifocal leukoencephalopathy; QALY, quality-adjusted life-year; SFDA, Saudi Food and Drug Authority; SPMS, secondary progressive MS; TB, tuberculosis; VZV, varicella-zoster virus.

## 3. Local Clinical Context: Domain One

The first domain asks what is actually known about the patients to whom a strategy would be applied. In the Thugbah community survey introduced in Section 1, the crude prevalence of MS was 0.04 per 1000 population [1]. Hospital-based series since then describe a disease more common, and more often diagnosed, than those early figures implied: in a cross-sectional study of 190 patients at a university hospital in Alkhobar, the mean age at onset was 26.3 years, mean disease duration was 6.4 years, and the female-to-male ratio was 1.6:1 [2]. At national level, the first multicentre Saudi MS registry, which by September 2018 had enrolled 2516 patients from 20 hospitals, reported a median age of 32 years and a female-to-male ratio of 2:1, and projected a national prevalence of 40.40 per 100,000 population, still lower than in several neighbouring Gulf countries [63]. Table 2 summarizes selected Saudi and regional cohorts with verified primary data.

The Alkhobar figures should be read with their limits in view. They derive from a single hospital-based centre, are subject to referral bias, and were not compared with a matched non-Saudi population; the study’s authors themselves described the clinical characteristics as not dissimilar to those reported in other Middle Eastern countries [2]. A mean onset in the mid-twenties and a relapsing-remitting predominance of about three-quarters do not, on their own, establish that Saudi patients differ from the populations enrolled in pivotal trials. Their practical relevance is more modest: they indicate that most patients seen in Saudi practice have relapsing disease, the setting in which the largest body of trial evidence exists, and that long treatment horizons make cumulative safety and affordability important considerations.

NMOSD requires separate comment because it is a distinct disease rather than a feature of MS. Optic neuritis and myelitis, the most frequent first presentations in the Alkhobar series [2], are also core manifestations of NMOSD, which is frequently misdiagnosed as MS [7]. We are not aware of comparative studies showing that NMOSD, or NMOSD-like presentations, are more frequent among Saudi patients than in European populations, and we make no such claim. The relevance of NMOSD to this framework is diagnostic: because the two conditions are treated differently, its exclusion is a prerequisite for MS therapy in any setting, and the capacity to exclude it reliably in the Kingdom is examined in Section 4.

Vitamin D deficiency is a recognized feature of Gulf populations [8], and low serum 25-hydroxyvitamin D has been associated with a higher risk of MS in prospective data [64]. Evidence that this exposure modifies MS outcomes in Saudi patients, or that it warrants a Saudi-specific supplementation strategy, is, however, lacking. The only Saudi interventional study we identified, a single-arm cohort of 35 RRMS patients given 10,000 IU cholecalciferol daily for 12 months, reported improvement in depressive symptoms that correlated inversely with serum vitamin D, but no improvement in the Expanded Disability Status Scale [65]. Such data are hypothesis-generating; they do not support disease-modifying claims, and the appropriate response is randomized controlled evaluation rather than locally tailored supplementation protocols.

Consanguinity is likewise a population characteristic rather than an MS phenotype. It is common in the Kingdom, and the Thugbah community survey recorded consanguineous marriage, particularly between first cousins, in 54.6% of couples [1]. No Saudi MS genetic study, however, has shown that consanguinity contributes to disease susceptibility, age at onset, or familial clustering, and any such mechanism remains speculative. Its relevance to this review lies in study design: family-structured recruitment and appropriate handling of relatedness will be needed if susceptibility and treatment response are to be studied in Saudi cohorts, a point taken up in Section 5.

Finally, the clinical burden has a service-level footprint. In a single-centre Jeddah cohort of 471 patients at King Abdulaziz Medical City, roughly one in four people with MS visited the emergency department at least once, most often for events directly attributable to the disease such as weakness and sensory disturbance [66]. Relapse-driven demand of this kind is a health-system fact, not only a clinical one, and it foreshadows the access and infrastructure determinants developed in the therapeutic sections that follow.

Taken together, the available Saudi data do not define a distinct MS phenotype, and nothing in them indicates that trial-derived efficacy or safety estimates should be discounted for Saudi patients. Within this framework, the first domain therefore functions mainly as a check: where no evidence of a clinically relevant population difference exists, trial evidence is used as the best available estimate [14,15], with the explicit caveat that its transferability to Saudi patients is presumed rather than demonstrated, and availability, affordability, monitoring requirement, and regional evidence are reported separately rather than combined into a single judgement.

**Table 2:** Selected Saudi and regional multiple sclerosis cohorts with verified primary data.

Study (First Author, Year)	Setting/Design	n	Key Findings Relevant to This Review
Al-Rajeh et al., 1993 [1]	Community-based neuroepidemiology survey, Saudi Arabia	Community sample	Early survey; MS reported as uncommon, consistent with limited case ascertainment of the period
Nazish et al., 2018 [2]	Cross-sectional, King Fahd Hospital of the University, Alkhobar	190	Mean age at onset 26.3 years; RRMS in 75%; female-to-male ratio 1.6:1; optic neuritis and myelitis most frequent first presentations
AlJumah et al., 2020 [63]	Nationwide multicentre MS registry, 20 hospitals across all regions	2516	Median age 32 years; female-to-male ratio 2:1; projected prevalence 40.40/100,000 population (61.95/100,000 Saudi nationals)
AlRuthia et al., 2021 [9]	Retrospective cost-effectiveness, tertiary centre, Riyadh	146	Drug classes pooled; monoclonal antibodies (rituximab/natalizumab) associated with lower odds of composite outcome (OR 0.17) at higher cost; oral agents less costly than interferon
Makkawi et al., 2024 [66]	Retrospective, King Abdulaziz Medical City, Jeddah	471	About one in four patients visited the ED at least once, most often for relapse-type events (weakness, sensory disturbance)
Elshony et al., 2026 [12]	Retrospective, Security Forces Hospital, Makkah	102	Off-label rituximab: NEDA-3 in 93.8% treatment-naive and 83.3% switch patients at 12 months; median follow-up 14 months; immunoglobulins not routinely monitored
Alroughani et al., 2021 (FINOMENA) [11]	Retrospective, uncontrolled before-after, 34 MENA centres incl. Saudi sites	806	Fingolimod: ARR fell 0.84 to 0.16; relapse-free rate rose 33.0% to 86.4%; NEDA-3 in 41.3%

All entries verified against the source abstract on PubMed. RRMS, relapsing-remitting MS; ARR, annualized relapse rate; NEDA, no evidence of disease activity; ED, emergency department; OR, odds ratio; MENA, Middle East and North Africa.

## 4. Diagnosis in Saudi Practice

Diagnosis is the point at which the clinical context and the Kingdom’s diagnostic infrastructure meet, and it is where gaps in infrastructure have the most immediate clinical consequences. The current international reference standard is the 2024 revision of the McDonald criteria [24], which superseded the 2017 revision [67]. The 2024 criteria add the optic nerve as a fifth anatomical location for dissemination in space; allow the central vein sign, paramagnetic rim lesions, and cerebrospinal fluid kappa free light chains to provide supportive evidence in specific situations; allow some cases of radiologically isolated syndrome to fulfil the criteria; apply a unified approach to relapsing and progressive presentations across the lifespan; and give guidance for people aged 50 years or older and those with comorbidities [24]. The Saudi Ministry of Health consensus on the diagnosis of MS in adults, published in 2023, recommends the 2017 criteria, while stressing that MRI findings alone are insufficient and that mimics, notably NMOSD and acute disseminated encephalomyelitis, must be ruled out [22]; the companion paediatric consensus applies the same criteria once these alternatives have been excluded [6]. That instruction matters in any setting, given the misdiagnosis risk described in Section 3, and its practical value in the Kingdom depends on the diagnostic infrastructure discussed below. Because the national consensus predates the 2024 revision, and because several of the new elements depend on resources beyond conventional MRI (susceptibility-sensitive MRI sequences for the central vein sign and paramagnetic rim lesions, kappa free-light-chain assays, and optic nerve assessment), adoption of the 2024 criteria in the Kingdom is itself a diagnostic-infrastructure question whose answer has not been documented.

**Figure 2:**
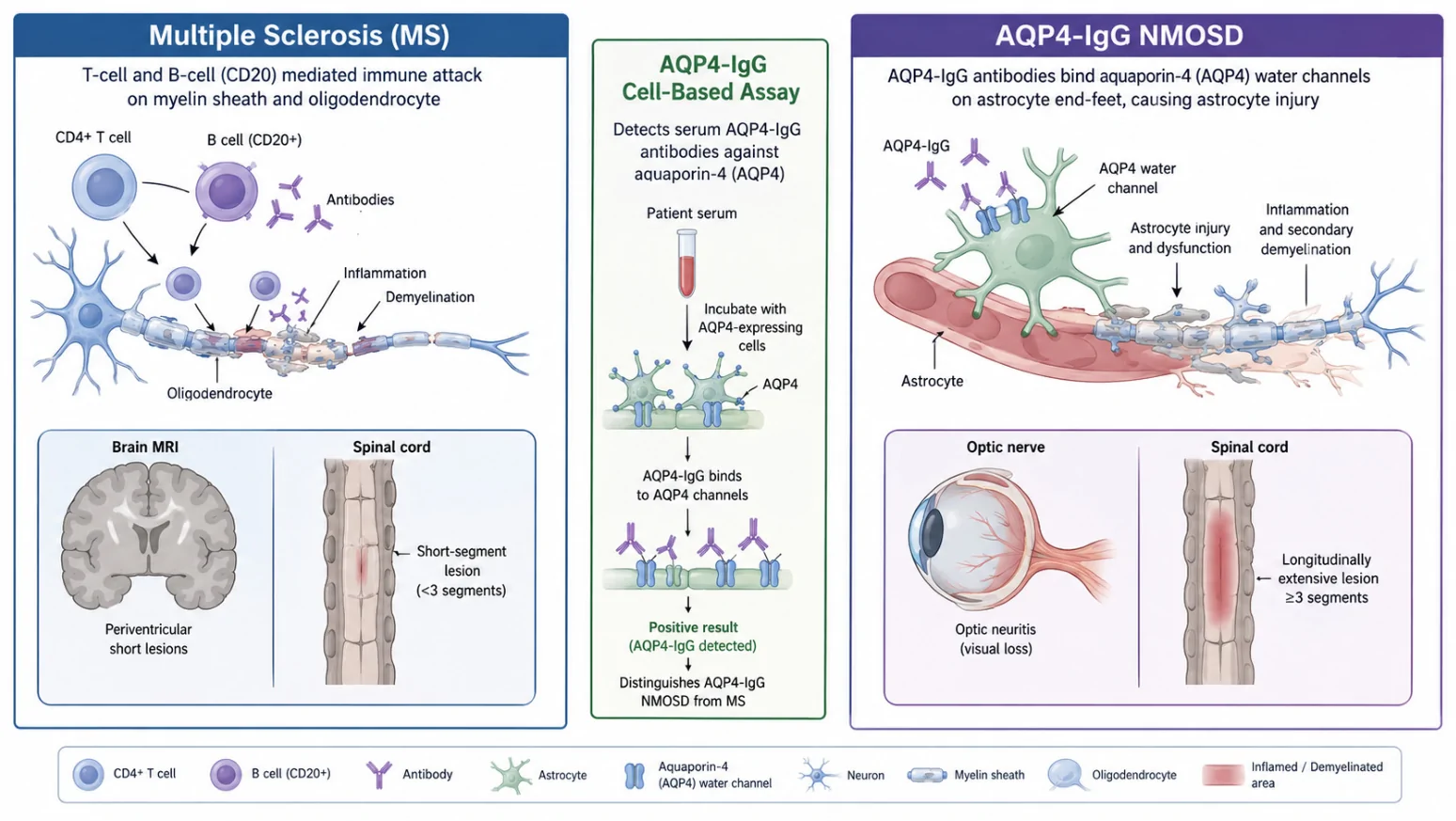
Distinguishing multiple sclerosis (MS) from AQP4-IgG neuromyelitis optica spectrum disorder (NMOSD). In MS (**left**), CD4+ T cells and CD20+ B cells drive an immune attack on the myelin sheath and oligodendrocyte, producing periventricular short brain lesions and short-segment (<3 vertebral segments) spinal cord lesions. In AQP4-IgG NMOSD (**right**), AQP4-IgG antibodies bind aquaporin-4 water channels on astrocyte end-feet, causing astrocyte injury and secondary demyelination, with optic neuritis and longitudinally extensive (≥3 segments) spinal cord lesions. The AQP4-IgG cell-based assay (**centre**) detects serum antibodies against aquaporin-4 and is the key test distinguishing the two conditions; accurate differentiation matters because some MS therapies may worsen NMOSD. Schematic based on [22,25,68,69]; for educational purposes, not to scale. Arrows indicate the direction of the depicted cellular interactions, antibody binding, and successive assay steps.

Antibody testing is the practical safeguard. The MENA regional consensus endorses the 2015 International Panel for NMO Diagnosis criteria [25] and places AQP4-IgG testing by cell-based assay at the centre of the diagnostic pathway, with MOGAD and ADEM as the differentials to weigh alongside MS [7,23]. The therapeutic stakes are high: in AQP4-IgG-positive disease, immunosuppressants such as azathioprine and mycophenolate, and the anti-CD20 agent rituximab, modify relapse risk and disability [68], whereas some MS therapies may worsen it; interferon beta, for example, has been associated with increased relapses and rising AQP4-IgG titres [69]. Table 3 and Figure 2 set out the features most useful for separating the two conditions in Saudi practice. Where a cell-based assay is available, the distinction can be made with confidence; where it is not, the diagnostic burden falls back onto clinical and imaging judgement, and the misclassification risk rises. How widely cell-based AQP4-IgG testing is available across Saudi centres has not, to our knowledge, been documented; physicians interviewed in a Saudi mixed-methods study did, however, describe limited access to MRI in smaller cities and a shortage of MS specialists in remote areas [10].

Conventional MRI, read against established lesion-pattern criteria such as the length of spinal cord lesions, remains the principal imaging aid in this distinction [22,25]. Quantitative morphometry is, at present, a research tool rather than a diagnostic test. A small retrospective study at King Saud University in Riyadh (25 patients with MS and 24 with NMOSD, analysed with the CAT12 toolbox) found the thalamus smaller in MS than in NMOSD at the group level [70]; a group difference of this kind, however, does not establish diagnostic accuracy, and no cut-off values, sensitivity, specificity, or validation in an independent cohort have been reported. On current evidence, thalamic volumetry therefore cannot be regarded as a clinical diagnostic adjunct. Optic neuritis, a frequent first event in this population, has its own diagnostic ambiguity; the international classification effort led by Petzold and colleagues—with Saudi contribution—was motivated in part by the reality that the cause and management of optic neuritis vary with geography, treatment availability, and ethnic background [71]. Blood-based markers, chiefly serum neurofilament light chain, track neuroaxonal injury, disease activity, and treatment effect in European cohorts [72], but they remain monitoring tools rather than diagnostic tests, and their availability in Saudi practice has not been documented.

On the evidence map, diagnosis shows why the domains are reported separately. The diagnostic knowledge is fully transferable; what is uncertain is the national distribution of the infrastructure—cell-based AQP4 assays, high-field MRI, and biomarker platforms—on which reliable exclusion of NMOSD depends, and the capacity needed for the newer elements of the 2024 criteria. The service-level pressure documented in the Jeddah emergency-department cohort, where relapse-type events drove a large share of visits, underscores that timely and correct diagnosis is not only a clinical goal but a system one [66].

**Table 3:** Distinguishing multiple sclerosis from AQP4-IgG neuromyelitis optica spectrum disorder: features of particular relevance to Saudi practice.

Feature	Multiple Sclerosis	AQP4-IgG NMOSD	Relevance to Saudi Practice
Serological marker	None specific	AQP4-IgG (cell-based assay)	Availability across centres not documented; where unavailable, misclassification risk rises
Optic neuritis	Often unilateral, milder	Often bilateral/severe, poor recovery	Optic neuritis a frequent first presentation in Saudi series; careful classification needed
Spinal cord lesion	Short-segment	LETM (≥3 vertebral segments)	MRI pattern aids differentiation when serology delayed
Brain/thalamus MRI	Periventricular, juxtacortical; thalamic atrophy	Area postrema, diencephalic; less thalamic atrophy	Group-level thalamic volume difference in one small Riyadh cohort; not a validated diagnostic test
First-line treatment	Disease-modifying therapies	Immunosuppression/biologics (e.g., anti-CD20, anti-C5, anti-IL6R)	Biologic choice regionally shaped by availability; conventional immunosuppressants when inaccessible
Drugs that may worsen disease	N/A	Some MS DMTs (e.g., interferon, natalizumab, S1P modulators)	Misdiagnosis carries direct therapeutic harm; confirm diagnosis before DMT
Diagnostic criteria	2024 McDonald criteria [24]	2015 IPND criteria	Saudi consensus predates the 2024 McDonald revision; IPND 2015 criteria adopted for NMOSD [7,22]

AQP4-IgG, aquaporin-4 immunoglobulin G; LETM, longitudinally extensive transverse myelitis; DMT, disease-modifying therapy. N/A, not applicable.

## 5. Molecular Targets and the Genetic-Architecture Question

**Figure 3:**
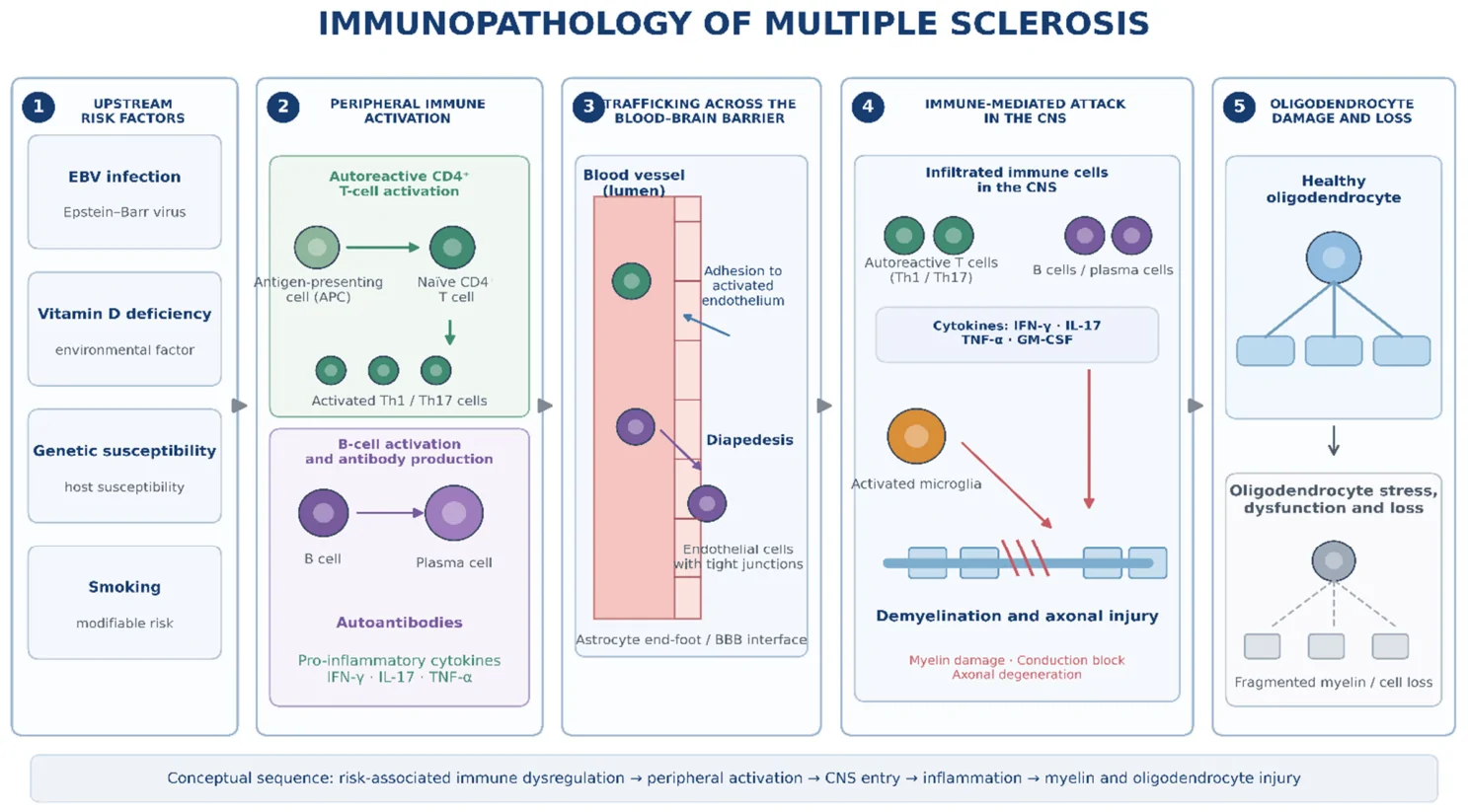
Immunopathology of multiple sclerosis. Upstream risk factors (Epstein-Barr virus infection, vitamin D deficiency, genetic susceptibility, and smoking) contribute to peripheral activation of autoreactive CD4+ T cells (Th1/Th17) and B cells, which release pro-inflammatory cytokines (IFN-gamma, IL-17, TNF-alpha) and autoantibodies. Activated immune cells adhere to and cross the blood–brain barrier by diapedesis. Within the central nervous system, infiltrating T cells, B/plasma cells, cytokines (including GM-CSF), and activated microglia mediate demyelination and axonal injury, culminating in oligodendrocyte stress, dysfunction, and loss. Schematic based on [64,73,74,75]; for educational purposes, not to scale.

Molecular biology is usually presented as settled at its foundation: the HLA-DRB1*15:01 allele within the major histocompatibility complex (MHC) is the best-known inherited risk factor, and genome-wide studies have mapped 200 autosomal susceptibility variants outside the MHC, 32 within the extended MHC, and one on chromosome X [73]. Established environmental and lifestyle risk factors, including Epstein–Barr virus infection, low vitamin D status, and smoking, act on this immunogenetic substrate, and some interact with HLA risk genes [74]; in a longitudinal cohort of more than 10 million US military personnel, MS risk increased 32-fold after Epstein–Barr virus infection [75]. The peripheral-to-central cascade these factors set in motion is summarized in Figure 3. For therapeutic purposes, these loci and pathways define where drugs can intervene: T-cell co-stimulation, B-cell survival, cytokine signalling, and the vitamin D endocrine axis.

The applicability question is whether this architecture, derived overwhelmingly from European-ancestry datasets, describes Saudi patients well enough to guide molecular-target-based treatment choices in the Kingdom. Current evidence suggests caution. The maps do not transfer cleanly. The 2026 GCC scoping review by Alsharoqi and colleagues synthesized the available HLA, non-HLA, vitamin D pathway, transcriptomic, and biomarker literature across Gulf populations and concluded that MS susceptibility in the region may be heterogeneous and not fully explained by the HLA-DRB1*15:01-centered model [8]. Their synthesis is candid about its own limits: fourteen studies met inclusion, only four yielded directly extractable effect estimates, and no pooled meta-analysis was possible because independent Gulf cohorts had not generated repeated variant-level data for most loci.

What the regional data do offer are leads rather than conclusions. Kuwaiti cohorts contribute the most extractable non-HLA and vitamin D pathway signals, including vitamin D receptor and vitamin D-binding protein associations; Bahraini work adds transcriptomic and inflammatory biomarker signals, among them IL-1RA, OASL, and TNFAIP6, that await prospective validation; Saudi contributions are mainly familial and registry-based, with exploratory mitochondrial studies as an additional layer [8]. Qatar, the UAE, and Oman remain sparsely represented in accessible primary genetic literature.

On the evidence map, molecular-target-guided therapy selection is an investigational treatment-selection approach (category E1) for Saudi practice, as it is internationally. The vitamin D axis is the partial exception: it is both a plausible therapeutic target and a documented population-level exposure in the Kingdom, which makes it the molecular avenue with the clearest rationale for regional research, although no controlled Saudi evidence yet links supplementation to MS outcomes (Section 3) [65]. For the remainder—HLA-guided risk stratification, non-HLA pharmacogenomic prediction of treatment response—the honest position is that the regional evidence base is too fragmented and too rarely replicated to support clinical use now. The corresponding research priority is concrete: harmonized Gulf registries with high-resolution HLA typing, standardized non-HLA genotyping, vitamin D phenotyping, and family-structured recruitment that can exploit, rather than be confounded by, the region’s consanguinity [8]. This review does not attempt to re-catalog that genetic literature, which the GCC scoping review has already mapped; our contribution is to record its clinical readiness for the Saudi therapeutic setting, deliberately, as investigational.

## 6. Established Disease-Modifying Therapy in the Kingdom: Access, Cost, and Off-Label Use

**Figure 4:**
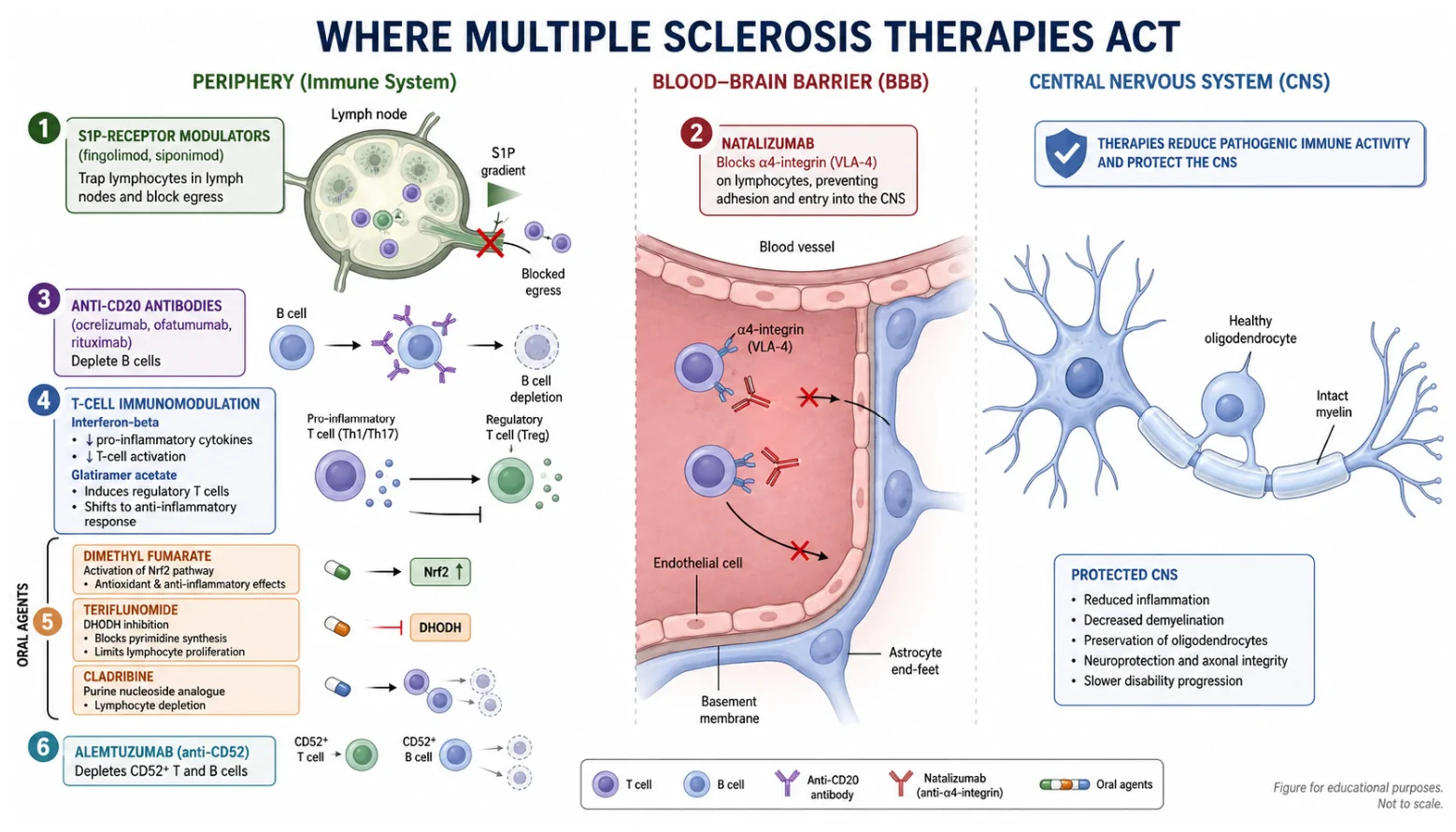
Sites of action of multiple sclerosis disease-modifying therapies across the periphery, the blood–brain barrier, and the central nervous system. In the periphery, S1P-receptor modulators (fingolimod, siponimod) trap lymphocytes in lymph nodes and block egress; anti-CD20 antibodies (ocrelizumab, ofatumumab, rituximab) deplete B cells; interferon-beta and glatiramer acetate modulate T-cell responses; oral agents act via Nrf2 activation (dimethyl fumarate), dihydro-orotate dehydrogenase inhibition (teriflunomide), and lymphocyte depletion (cladribine); and alemtuzumab depletes CD52+ T and B cells. At the blood–brain barrier, natalizumab blocks alpha4-integrin (VLA-4), preventing lymphocyte entry into the central nervous system. Collectively these therapies reduce pathogenic immune activity and protect the central nervous system. Schematic based on the agents and pivotal trials cited in Table 4 [26,29,31,33,35,36,40,43,45,49,52,53]; for educational purposes, not to scale. Arrows indicate the depicted cellular or molecular effects; red crosses and blunt-ended lines indicate blockade or inhibition.

On paper, Saudi neurologists prescribe from the same disease-modifying menu as their European counterparts. In practice, physicians report that the choice is also shaped by cost, drug availability, and insurance formularies [10], although the extent of these constraints has not been quantified nationally. The approved menu spans injectable immunomodulators—interferon beta and glatiramer acetate [26,29]—oral agents including dimethyl fumarate, teriflunomide, and cladribine [31,33,40], and high-efficacy monoclonals; their sites of action are illustrated in Figure 4, and their mechanisms, monitoring demands, and regional evidence are collected in Table 4.

Saudi cost-effectiveness data are limited to a single study. A retrospective analysis at one university-affiliated tertiary centre in Riyadh (*n* = 146 RRMS patients; 40 on oral agents, 66 on interferon-based therapy, and 40 on monoclonal antibodies) compared drug classes using electronic-health-record outcomes, adjusting for age, sex, and duration of therapy [9]. Monoclonal antibodies were associated with lower odds of a composite outcome of relapse, new MRI lesions, and disability progression (OR 0.17, 95% CI 0.068–0.428), at an additional annual cost of about USD 5718 relative to oral agents, and oral agents were associated with lower cost than interferon beta-1a. These estimates do not show that one class is superior or that oral therapy is economically favoured in Saudi practice: the analysis pooled pharmacologically distinct drugs (rituximab with natalizumab; fingolimod with dimethyl fumarate and teriflunomide), was small and single-centre, and is open to confounding by indication, and its authors called for more robust study designs. For patients facing long treatment horizons, cost data of this kind are nonetheless relevant to treatment planning. Cladribine, an oral immune-reconstitution therapy, has been modelled as cost-effective from the Saudi payer perspective in an analysis with manufacturer involvement [42] and appraised for regional practice [76].

**Figure 5:**
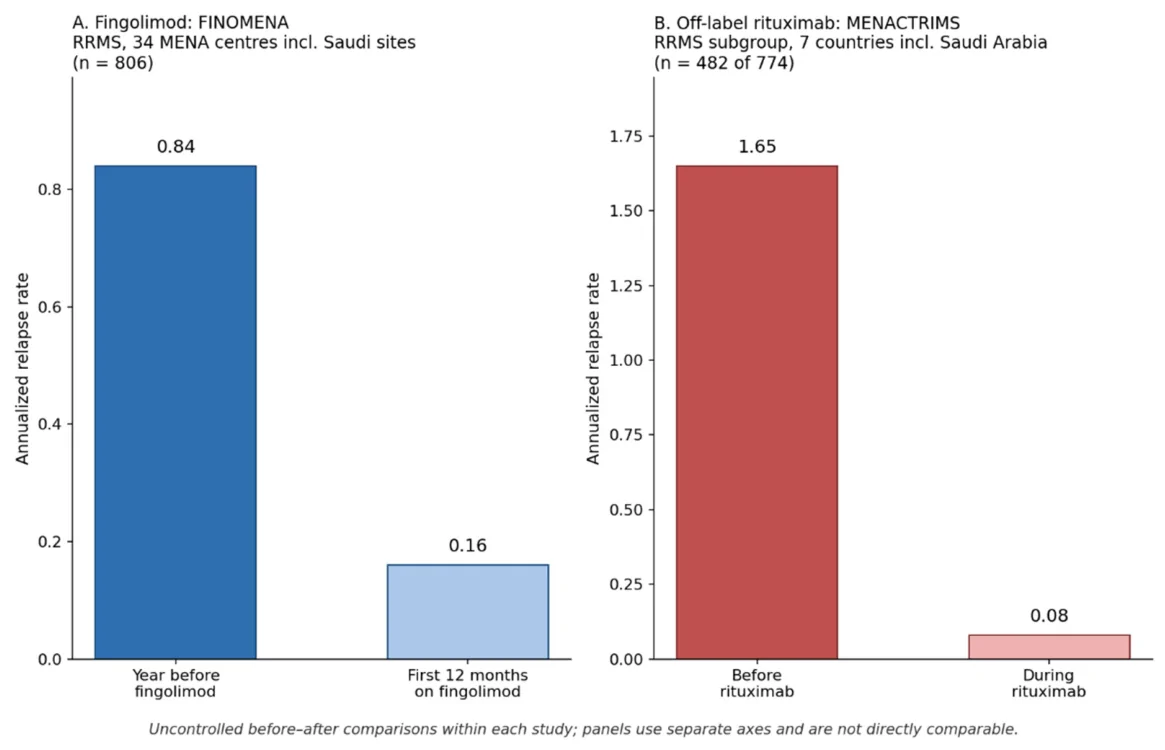
Annualized relapse rate (ARR) before and during treatment in two regional observational studies, shown as separate panels. (**A**) Fingolimod: retrospective FINOMENA study across 34 Middle East and North Africa centres including Saudi sites (*n* = 806 patients with relapsing-remitting MS); ARR in the year before fingolimod initiation versus the first 12 months of treatment [11]. (**B**) Off-label rituximab: retrospective MENACTRIMS registry analysis across seven countries including Saudi Arabia; values are for the relapsing-remitting subgroup (*n* = 482 of 774 patients), comparing ARR before rituximab with ARR during treatment [16]. Each panel is an uncontrolled before–after comparison within a single study; the panels use separate axes, differ in population, baseline activity, and follow-up, and must not be read as a comparison between the two therapies.

Off-label rituximab illustrates both the value and the limits of the regional evidence. Rituximab is increasingly used off-label for MS, particularly where access to approved B-cell-depleting therapies is limited [12], but how often it is prescribed in Saudi Arabia, and how far its use reflects formulary constraints rather than clinical preference, has not been documented; in national registry data from 2015 to 2018 it accounted for fewer than 1% of recorded DMT prescriptions, which were dominated by interferons [10]. Two recent observational studies report its outcomes. In a single-centre cohort at Security Forces Hospital in Makkah (*n* = 102) [12], NEDA-3 was achieved at 12 months in 93.8% of treatment-naïve and 83.3% of switch patients, with mild infusion reactions in 2.9%; the authors note the single-centre retrospective design, a median follow-up of 14 months, and the absence of routine immunoglobulin monitoring. In the MENACTRIMS registry across seven countries including Saudi Arabia (*n* = 774, of whom 482 had RRMS), the annualized relapse rate in the RRMS subgroup fell from 1.65 to 0.08 (Figure 5) and NEDA-3 was achieved in 47.3% of RRMS patients, with predominantly mild infusion-related adverse events [16]. These uncontrolled before-and-after comparisons are consistent with the efficacy of B-cell depletion established in randomized trials (Section 7), but they cannot establish the comparative effectiveness of rituximab or define a treatment protocol.

Physician-reported data point in the same direction but are limited. In a sequential explanatory mixed-methods study combining national registry data with interviews of nine neurologists, participants identified drug availability, insurance-formulary restrictions, access to MRI in smaller cities, and the shortage of MS specialists in remote areas as challenges in MS care, and the authors concluded that MS drugs were expensive and unavailable in most hospitals, calling for cooperative procurement [10]. Because these are qualitative findings from a small sample, together with registry data ending in 2018, they indicate that access constraints exist rather than their current magnitude. During the COVID-19 pandemic, Saudi expert consensus likewise placed tolerability and safety at the centre of DMT selection [4].

Cost evidence from the Kingdom is more substantial than access evidence, but it measures something different. A single-centre cost-of-illness analysis from the public-payer perspective (*n* = 193; 2019–2020) estimated annual direct medical costs of about USD 21,541 per patient, with disease-modifying therapy accounting for 74% of the total [77]. A Markov model using Riyadh hospital costs found that none of the interferon or oral agents modelled was cost-effective at a willingness-to-pay threshold of USD 100,000 per quality-adjusted life-year [27], and a two-centre Riyadh analysis estimated the incremental cost of natalizumab over rituximab at about USD 35,383 per patient, with a 59% probability that rituximab was dominant [44]. In a Delphi consensus of Saudi MS experts, panellists reported that high-efficacy therapies prescribed in the private sector carry a risk of insurance claims being denied, and some described JC virus testing as logistically burdensome [41]. Taken together, these studies show that disease-modifying therapies are a major cost to the Saudi payer and that cost- and logistics-related constraints are reported by clinicians. They do not show how often patients are denied a preferred therapy, and no Saudi study has compared the relative weight of cost, infrastructure, and other barriers. We therefore treat these factors as documented constraints of unquantified magnitude, not as established main barriers to care.

On the evidence map, the established DMTs fall in category A: each has phase 3 randomized evidence and is listed in the Saudi consensus recommendations [20]. Their affordability and monitoring domains differ substantially (Table 1), and the regional effectiveness data that inform them are observational. Saudi cost data indicate that high-efficacy monoclonal antibodies carry higher costs than oral or injectable agents [9,44]; whether this translates into restricted access for individual patients has not been measured.

**Table 4:** Disease-modifying therapies for multiple sclerosis: mechanism, administration, key monitoring, and pivotal or regional evidence relevant to the Saudi setting.

Therapy	Mechanism/Target	Route	Key Monitoring	Pivotal/Regional Evidence
Interferon beta-1a	Immunomodulation of T-cell activation and cytokine profile	SC/IM	Liver tests and blood counts at 1, 3 and 6 months, then periodically; thyroid tests; depression [28]	PRISMS: relapse-rate reduction 27–33% vs. placebo [26]
Glatiramer acetate	Immune deviation toward regulatory Th2 responses	SC	No routine laboratory monitoring; injection-site reactions, lipoatrophy; anaphylaxis (boxed warning) [30]	Copolymer-1: 32% fewer relapses vs. placebo (~30 mo) [29]
Dimethyl fumarate	Nrf2 pathway activation; anti-inflammatory/cytoprotective	PO	Blood count with lymphocytes before treatment, at 6 months, then every 6–12 months; liver tests; PML risk with lymphopenia [32]	DEFINE: significant relapse and MRI benefit [31]; lower cost than interferon in one Saudi centre [9]
Teriflunomide	Dihydro-orotate dehydrogenase inhibition; limits lymphocyte proliferation	PO	ALT at least monthly for 6 months; blood count; latent TB screening; blood pressure; teratogenic: pregnancy exclusion, contraception, accelerated elimination [34]	TEMSO: reduced ARR and disability [33]
Fingolimod	Sphingosine-1-phosphate receptor modulation; lymphocyte sequestration	PO	First-dose cardiac, macular OCT, VZV serology	FREEDOMS: ARR 0.18 vs. 0.40 [35]; MENA real-world FINOMENA *n* = 806 [11]
Siponimod	Selective S1P1/S1P5 modulation	PO	First-dose cardiac, macular, CYP2C9 genotype	EXPAND (SPMS): reduced disability progression [36]
Cladribine	Selective lymphocyte depletion (immune reconstitution)	PO (short courses)	Lymphocyte counts, infection/malignancy surveillance	CLARITY: reduced relapse and MRI activity [40]; KSA cost-effectiveness [42]; regional guidance [76]
Natalizumab	Anti-alpha4-integrin; blocks lymphocyte CNS entry	IV/SC	JCV serostatus, PML vigilance	AFFIRM: high efficacy [49]; MENACTRIMS registry *n* = 1954 [13]; Saudi PML case after natalizumab-to-fingolimod switch [51]
Ocrelizumab	Anti-CD20 B-cell depletion	IV	Infusion reactions, immunoglobulins, infections	OPERA I/II vs. interferon [43]; Saudi access not quantified
Ofatumumab	Anti-CD20 B-cell depletion (fully human)	SC (self-injection)	Immunoglobulins, infections	ASCLEPIOS I/II vs. teriflunomide [45]; unmet-needs positioning in KSA [46]
Rituximab (off-label)	Anti-CD20 B-cell depletion	IV	Immunoglobulins, infusion reactions, infections	Observational KSA/MENA cohorts: *n* = 102 [12]; *n* = 774, 482 RRMS [16]
Alemtuzumab	Anti-CD52 depletion of T and B cells	IV (annual courses)	Autoimmunity (thyroid, ITP), infections; long monitoring	CARE-MS I first-line [52]; CARE-MS II relapse on prior therapy [53]
BTK inhibitors (e.g., tolebrutinib)	Bruton tyrosine kinase inhibition; B-cell and innate CNS compartment	PO	Drug-induced liver injury; intensive early liver-function monitoring	Phase 3: not superior to teriflunomide in relapsing MS; reduced disability progression in nrSPMS [54,55]; no regional data

SC, subcutaneous; IV, intravenous; PO, oral; JCV, JC virus; PML, progressive multifocal leukoencephalopathy; ARR, annualized relapse rate; RRMS, relapsing-remitting MS; SPMS, secondary progressive MS.

## 7. Anti-CD20 and High-Efficacy Immune-Pathway Therapy: Regional Evidence and Its Limits

B-cell depletion has become the high-efficacy backbone of MS treatment worldwide, and it is where access constraints, if present, would matter most. The approved anti-CD20 agents—ocrelizumab and ofatumumab—each established high efficacy against active relapsing disease in pivotal trials [43,45], and are relevant to relapsing disease, the predominant course reported in Saudi series [2]; an expert-panel analysis with manufacturer involvement positioned ofatumumab as an option in Saudi practice, particularly for patients who would benefit from early high-efficacy therapy or who prefer the convenience of self-injection, and suggested that comparative cost data could support its broader inclusion in formularies [46]. Whether reimbursement limits the use of approved anti-CD20 agents in the Kingdom has not been systematically documented; off-label rituximab is also used in regional practice (Section 6) [12,16]. Ublituximab, a further anti-CD20 antibody authorized in the European Union in May 2023, after the Saudi consensus was issued [48], reduced the annualized relapse rate relative to teriflunomide in the phase 3 ULTIMATE I and II trials [47]; we did not assess its Saudi registration status.

Where the choice is between B-cell depletion and α4-integrin blockade—the latter established as a high-efficacy option in the AFFIRM trial [49]—regional data now speak directly. A propensity-score-matched analysis of the MENACTRIMS registry across seven Middle Eastern countries including Saudi Arabia (*n* = 1954; 768 on rituximab, 509 on ocrelizumab, 677 on natalizumab) found natalizumab associated with a lower annualized relapse rate than anti-CD20 therapy (0.062 vs. 0.092, *p* = 0.001) and a higher rate of disability improvement (9.3% vs. 5.5%, *p* = 0.03), while confirmed disability progression, MRI outcomes, and NEDA-3 were comparable between groups [13]. Adverse events were more frequent with anti-CD20 (36.4% vs. 27.5%, *p* = 0.001), though natalizumab showed lower treatment persistence—a trade-off that maps onto the safety concern that shadows natalizumab everywhere. As a retrospective registry-based comparison, this analysis remains open to residual confounding despite propensity-score matching, and it complements rather than replaces randomized evidence.

Alemtuzumab, an anti-CD52 depleting antibody given as annual courses, offers durable efficacy but carries a distinctive secondary-autoimmunity risk that demands prolonged surveillance [52,53]; in a Delphi consensus of Saudi experts it was ranked as carrying the highest administration and monitoring burden of the high-efficacy DMTs assessed [41]. A related long-term-safety concern shadows natalizumab: progressive multifocal leukoencephalopathy is an infrastructure question as much as a pharmacological one. Safe natalizumab use depends on JC virus serostatus surveillance and prompt recognition of PML, which is not always straightforward: a Saudi case report documented PML, in a JC virus antibody-positive patient switched from natalizumab to fingolimod, that developed without the typical radiological changes, a presentation that can delay diagnosis in any setting and demands both vigilance and access to confirmatory testing [51]. Prescribing patterns in the Kingdom have already shown sensitivity to such considerations; during the COVID-19 pandemic, international MSBase registry data, to which a Saudi centre contributed, recorded shifts toward natalizumab and cladribine and away from fingolimod and, transiently, anti-CD20 therapy, a reminder that perceived safety, not efficacy rankings alone, moves real-world prescribing [78]; regional guidance addressed vaccination and DMT timing through the same period [79].

Bruton tyrosine kinase inhibition, the newer entrant, illustrates a different evidence status within this same therapeutic family. Its evidence base has, however, moved well beyond the phase 2 stage [80]. In the phase 3 GEMINI 1 and 2 trials in relapsing MS, tolebrutinib was not superior to teriflunomide in reducing the annualized relapse rate [55], whereas in the phase 3 HERCULES trial in non-relapsing secondary progressive MS it reduced 6-month confirmed disability progression relative to placebo (22.6% vs. 30.7%; hazard ratio 0.69, 95% CI 0.55–0.88) [54]. Across the phase 3 programme, which also included primary progressive MS (PERSEUS), where tolebrutinib showed no meaningful benefit, drug-induced liver injury emerged as an important identified risk: among 2958 exposed participants, 4.7% had alanine aminotransferase elevations above three times the upper limit of normal and six met Hy’s law criteria, one of whom died after liver transplantation, with all cases occurring within the first 90 days of exposure, and enhanced early liver-test monitoring was introduced to mitigate the risk [57]. Regulatory decisions have diverged. The US Food and Drug Administration issued a complete response letter for non-relapsing secondary progressive MS in December 2025 [81], whereas the European Commission approved tolebrutinib in June 2026 for secondary progressive MS without relapses in the preceding two years, and the manufacturer reports approval for this population in the United Arab Emirates [56]. Tolebrutinib is therefore no longer only a pipeline agent, but its first approved indication lies in non-relapsing progressive disease rather than in relapsing MS, where the available alternatives are already established. For Saudi practice, its position depends on national regulatory review, for which we identified no publicly available decision at the time of revision, on the capacity to deliver intensive early liver-function monitoring, and on the generation of regional real-world data, of which there are none so far; it remains a regulatory and research question rather than a treatment recommendation. On the evidence map, ocrelizumab and natalizumab fall in category A, ofatumumab and ublituximab in category C, rituximab in category B, and tolebrutinib in category D. All infused agents carry a tier 3 monitoring requirement; for natalizumab this includes JC virus antibody-index risk stratification [50], which the Saudi consensus uses to guide switching [20], and for which Saudi data now document a practical gap: in a Riyadh cohort of 91 patients considered for natalizumab, 39.6% were anti-JC virus seropositive, only 4 of the 83 patients who received natalizumab had been tested before initiation, and the median laboratory turnaround time was 20 days [19]. The national distribution of testing capacity remains undocumented. Fingolimod carries the same tier (Section 8), consistent with the equal ranking of the monitoring burden of the two drugs by a Saudi Delphi panel [41].

## 8. Sphingosine-1-Phosphate Receptor Modulation: Regional Real-World Evidence

Sphingosine-1-phosphate (S1P) receptor modulators sequester lymphocytes in secondary lymphoid organs, reducing their egress into the circulation and, in turn, into the central nervous system. Two features make this class distinctive for the Saudi setting. It is oral, which removes the need for infusion services, a potential practical advantage given the reported limits on specialist access outside major cities [10]. And fingolimod has one of the larger regional real-world datasets among the agents examined here.

That evidence comes from FINOMENA, a retrospective study of fingolimod in relapsing-remitting disease across 34 centres in the Middle East and North Africa, including Saudi sites (*n* = 806) [11]. Over twelve months, the relapse-free rate rose from 33.0% to 86.35%, the annualized relapse rate fell from 0.84 to 0.16 (Figure 5), mean EDSS declined from 2.69 to 2.01, and the proportion of patients with gadolinium-enhancing lesions dropped from 57.84% to 12.93%; NEDA-3 was reached in 41.3%, and 99.38% remained on treatment beyond a year. Adverse events occurred in 16.13%, mostly lymphopenia and leukopenia, with serious events in 1.61%. These are uncontrolled before-and-after data: most patients (79.7%) had received a prior DMT, there was no comparator arm, and a manufacturer employee was among the authors, so the study shows that outcomes in regional practice are consistent with the pivotal trial evidence discussed below rather than providing independent evidence of efficacy.

Fingolimod’s efficacy was established in the placebo-controlled FREEDOMS trial [35] before this regional experience accrued. Its selective successors—ozanimod, ponesimod, and siponimod—share the oral-access advantage but not its regional evidence; siponimod, for instance, slowed disability progression in secondary progressive disease in the EXPAND trial [36], yet its Gulf real-world footprint is still minimal, a gap that the framework records as a regional research priority rather than as a reason to discount trial efficacy; ozanimod and ponesimod likewise showed lower relapse rates than active comparators in phase 3 trials [37,38,39]. Deployment of S1P modulators carries monitoring obligations—first-dose cardiac observation (universal for fingolimod; dose titration and conditional observation for the newer agents), macular assessment, and varicella-zoster status—that add a service requirement worth counting in access terms. On the evidence map, fingolimod and siponimod fall in category A and ozanimod and ponesimod in category C, a difference that reflects listing in the Saudi consensus [20] rather than efficacy. Fingolimod’s oral route removes the need for infusion services, but its observed first-dose cardiac monitoring and macular assessment place it in monitoring tier 3, the same tier as natalizumab; its larger regional dataset is reported descriptively and does not change its category.

## 9. Emerging and Experimental Modalities: A Regional Research Agenda

The therapies discussed so far can, in some form, reach a Saudi patient today. The modalities in this section cannot. Each is scientifically interesting and, in most cases, still preclinical or early-phase even globally; none has generated Saudi or Gulf data. Rather than survey them as though regional applicability were imminent, we treat them as a research agenda and state, for each, what the Kingdom would need to build before the question of applicability becomes real. These modalities share an evidence status, not a purpose, and the distinction matters for what the Kingdom would need first. Exosome-based biomarkers, like the molecular-target selection discussed in Section 5, are treatment-selection approaches (category E1), which require analytical and clinical validation of a test before any effect on patient outcomes can be studied. NK-cell modulation, dopaminergic immunomodulation, and plant-derived compounds are candidate therapeutic agents (E2), which require the conventional sequence of phase 1 to phase 3 trials. CRISPR-based editing and exosome-based delivery are enabling technologies (E3), which are at present research tools or delivery platforms rather than treatments. The subsections below state the prerequisite for each.

### 9.1. Natural Killer Cell Modulation

NK-cell biology appears altered in MS: in a small study of 12 relapsing-remitting patients and 11 controls, patients showed reduced exercise-induced redeployment of NK cells and lower expression of a migration-related chemokine receptor, which has been read as an immunoregulatory mechanism that might be therapeutically enhanced [58]. The approach remains investigational. No study has characterized NK subset distribution in Saudi MS patients; such descriptive characterization would be the logical first step before any therapeutic question can be posed regionally.

### 9.2. CRISPR-Based and Gene-Directed Strategies

In MS, CRISPR-Cas9 has so far served as a laboratory tool—used, for example, to dissect pathogenic astrocyte responses in experimental models—rather than as a therapy [59]. Its regional relevance is contingent on a prerequisite the Kingdom does not yet have: a validated, Gulf-specific genetic map. Because Western HLA- and locus-based models may not transfer intact to Arabian Gulf ancestry, any future gene-directed intervention would have to rest on regional genetic data that the current fragmented evidence cannot supply—which makes registry-linked genotyping, not gene editing, the actionable near-term priority.

### 9.3. Dopaminergic Immunomodulation

Dopamine receptors expressed on T cells and antigen-presenting cells can shift the Th17–regulatory T-cell balance, raising the possibility of repurposing dopaminergic agents [60]. The evidence is preclinical, and there is no regional work. The realistic Saudi contribution here is exploratory and mechanistic rather than clinical.

### 9.4. Plant-Derived and Natural-Product Interventions

Several plant-derived compounds show activity in experimental autoimmune encephalomyelitis models; a synthetic phenethyl ester derivative of rosmarinic acid, a polyphenol abundant in Lamiaceae herbs, for instance, reduced the encephalitogenic cytokines IFN-γ and IL-17 and lessened disease severity in rats [61]. The extent to which Saudi patients with MS use herbal or traditional products alongside prescribed DMTs has not, to our knowledge, been studied. Establishing it is a prerequisite for two regional priorities—controlled clinical evaluation of the most promising candidates, and pharmacovigilance for herb–drug interactions in patients on immunomodulatory therapy, a risk that has not been assessed in this population.

### 9.5. Exosome-Based Diagnostics and Delivery

Exosomes have been proposed both as sources of diagnostic and prognostic biomarkers, such as exosomal microRNAs, and as vehicles for delivering therapeutic cargo across the blood–brain barrier [62], but this work remains largely experimental. For the Kingdom, exosome research is gated by the same infrastructure deficit that limits molecular and genetic work: it depends on harmonized biobanking and sample-handling capacity whose current extent in Saudi centres has not been described. Exosomes therefore belong to the same institutional priority as the genetic agenda—biobank and registry infrastructure first, translational application later.

All five are investigational (category E), as they are internationally, but they fall into different subgroups with different next steps: E1 (exosome-based biomarkers), E2 (NK-cell modulation, dopaminergic immunomodulation, plant-derived products), and E3 (CRISPR-based strategies, exosome-based delivery). For each, the deliverable is a defined research priority rather than a recommendation.

## 10. Conclusions

Multiple sclerosis in Saudi Arabia has moved from an under-recognized condition to a substantial cause of neurological disability in young adults, but the available Saudi data do not establish a distinct clinical phenotype that would invalidate trial-derived evidence, although the transferability of that evidence has been presumed rather than formally tested. How far global advances reach Saudi patients therefore depends on current availability (which has not been documented), affordability, monitoring capacity, and regional evidence, which we have mapped separately for each strategy rather than combined into a single grade.

The picture that emerges is uneven in an instructive way. Established disease-modifying therapies are listed in the 2022 Saudi consensus recommendations, although their current registration, supply, and reimbursement have not been documented, and their use is shaped by factors beyond efficacy: physicians report cost, availability, and insurance formularies as constraints on treatment choice, off-label rituximab is used alongside approved high-efficacy agents, and regional observational data for fingolimod, natalizumab, and rituximab are consistent with pivotal trial results without establishing comparative effectiveness. Natalizumab remains a high-efficacy option whose safe use depends on JC virus and progressive multifocal leukoencephalopathy monitoring capacity, the national distribution of which has not been documented. Diagnosis is a case in which the knowledge transfers fully, now under the 2024 McDonald revision, but the national distribution of the infrastructure—cell-based AQP4 testing, MRI, and safety monitoring—is undocumented, even though reliable exclusion of NMOSD depends on it. Among investigational strategies, molecular-target-guided therapy and the experimental modalities of gene editing, NK modulation, dopaminergic repurposing, natural products, and exosome-based approaches have no regional evidence base at all.

The recurring finding is that, for most strategies, the information missing for the Saudi setting is not clinical efficacy but data on cost and access, monitoring capacity, and regional outcomes. Saudi studies document substantial costs of disease-modifying therapy to the payer [27,77], and clinicians report formulary and logistic constraints [10,41], but no study has measured how often these constraints alter treatment or ranked them against other barriers. A regional research agenda follows directly from the framework and is set out in Table 5. Priorities include validated national prevalence and outcome tracking through linked registries; Gulf-specific genetic and vitamin D pathway characterization that exploits rather than ignores consanguinity; controlled evaluation of high-value, access-appropriate therapies including off-label rituximab, whose current share of prescribing is unknown; pharmacovigilance for both immunomodulatory safety and herb–drug interactions; and biobanking infrastructure to make biomarker and molecular research possible. Measuring these constraints, and building the local evidence and infrastructure needed to address them, is the practical agenda that follows from the map.

## Figures and Tables

**Table 5:** Prioritized regional research and infrastructure agenda for multiple sclerosis in Saudi Arabia, derived from the evidence map.

Domain	Current Gap in the Kingdom	Priority Action	Domain Addressed
Epidemiology & outcomes	No unified national prevalence/outcome tracking	Linked national MS registry with standardized outcome capture	Clinical suitability (local context); regional evidence
Genetics	Fragmented, rarely replicated Gulf variant data; Western HLA model may not transfer	Harmonized Gulf genotyping with high-resolution HLA and family-structured recruitment	Regional evidence
Vitamin D	Widely reported population deficiency; no controlled Saudi MS outcome data	Randomized controlled trials with relapse, MRI, and disability endpoints	Clinical suitability (local context); regional evidence
Therapeutic access	Formulary/insurance constraints reported; off-label rituximab in use, extent unknown	Prospective evaluation and pharmacovigilance of the off-label pathway already in use	Availability; affordability
Affordability and access measurement	Payer costs documented [27,44,77]; how often patients are denied or switched from a preferred therapy not measured	Registry-linked measurement of prescribing, insurance denials, treatment switches and out-of-pocket costs, and studies ranking barriers to care	Affordability; availability
Current national availability	Registration, supply, formulary inclusion, and reimbursement of each DMT not publicly documented; 2022 consensus predates newer agents	Periodic national audit of SFDA registration, institutional formulary listing, and reimbursement for each DMT, linked to the national registry	Availability
Transferability of trial evidence	No Saudi-specific subgroup analyses of pivotal trials; transferability presumed, not demonstrated	Registry-based comparison of real-world effectiveness and safety against pivotal-trial benchmarks; pharmacogenomic (e.g., CYP2C9) and anti-JC virus serostatus profiling	Clinical suitability; regional evidence
Monitoring infrastructure	Distribution of AQP4 testing, JCV/PML monitoring, and MRI capacity undocumented	National mapping of diagnostic and safety-monitoring capacity, followed by targeted investment	Monitoring requirement
Natural products	Extent of herbal use alongside DMTs and herb–drug interactions not studied	Controlled evaluation plus interaction pharmacovigilance in patients on DMTs	Clinical suitability; regional evidence
Biobanking	Limited harmonized sample handling for molecular/exosome research	National biobank infrastructure to enable biomarker and molecular studies	Monitoring requirement; regional evidence

DMT, disease-modifying therapy; HLA, human leukocyte antigen.

## Data Availability

No new data were created or analyzed in this study. Data sharing is not applicable to this article.
